# Favorable Pregnancy Outcomes in Women With Well-Controlled Pulmonary Arterial Hypertension

**DOI:** 10.3389/fmed.2021.689764

**Published:** 2021-07-05

**Authors:** Nadine Corbach, Charlotte Berlier, Mona Lichtblau, Esther I. Schwarz, Fiorenza Gautschi, Alexandra Groth, Rolf Schüpbach, Franziska Krähenmann, Stéphanie Saxer, Silvia Ulrich

**Affiliations:** ^1^Department of Pulmonology, University Hospital Zurich, Zurich, Switzerland; ^2^Department of Anesthesiology, University Hospital Zurich, Zurich, Switzerland; ^3^Department of Gynacology, University Hospital Zurich, Zurich, Switzerland

**Keywords:** pulmonary hypertension, pulmonary arterial hypertension, pregnancy, outcome, delivery

## Abstract

**Introduction:** Since pregnancy in women with pulmonary arterial hypertension (PAH) is associated with a high risk of morbidity and mortality, it is recommended that pregnancy should be avoided in PAH. However, some women with mild PAH may consider this recommendation as unsuitable. Unfortunately knowledge on pregnancy outcomes and best management of PAH during pregnancy is limited.

**Methods:** Data from all women with PAH who were followed during pregnancy by a multidisciplinary team at a tertiary referral center for PAH and who delivered between 2004 and 2020 were retrospectively analyzed in a case series. PAH risk factor profiles including WHO functional class (WHO-FC), NT-pro-BNP, echocardiographic pulmonary arterial pressure (PAP) and right heart function were analyzed prior to, during and following pregnancy.

**Results:** In seven pregnancies of five women with PAH (median age 29 (27; 31) years), there were no abortions or terminations. Five pregnancies were planned (all in WHO-FC I-II), two incidental (WHO-FC II, III). During pregnancy none of the women had complications or clinical worsening of PAH. After a median pregnancy duration of 37 1/7 weeks all gave birth to healthy babies by cesarean section in spinal anesthesia. During pregnancy, PAP tended to increase, whilst the course of WHO-FC and NT-pro-BNP were variable and no trend could be detected.

**Conclusion:** Women with PAH with a low risk profile closely followed by a multidisciplinary team had a favorable course during and after pregnancy, resulting in successful deliveries of healthy newborns.

## Introduction

Pulmonary arterial hypertension (PAH) is a relatively rare condition with exertional dyspnea being the main symptom (1). Diagnosis of PAH requires evidence of precapillary pulmonary hypertension by right heart catheterization (RHC) that is defined by a mean pulmonary artery pressure (PAP) >20 mmHg and a pulmonary artery wedge pressure <15 mmHg along with a pulmonary vascular resistance (PVR) >3 WU at rest (2). Patients in whom mean PAP decreases by >10 mmHg below 40 mmHg during vasoreactivity testing, are classified as vasoreactive. Prognosis is more favorable in patients with PAH with a positive response to vasoreactivity testing (1, 3). After confirmation of PAH diagnosis in an expert center, timely initiation of often combined therapy is warranted in non-vasoreactive PAH. Rather high doses of calcium channel blockers (CCB) are used in vasoreactive patients. Supportive therapy as well as counseling patients concerning their disease and general measures are important as combined approach with the drug therapy (4). A cornerstone of counseling women with PAH of childbearing age is discussing contraception, family planning and the risks of pregnancy (5). It is generally recommended that pregnancy should be avoided in patients with PAH, particularly in persistently compromised pulmonary hemodynamics (1, 6). Physiological changes associated with pregnancy and puerperium pose a great challenge on the cardiovascular system including large increases in blood volume and oxygen consumption (7, 8). Patients with PAH have a limited ability to compensate this physiologic changes and to increase in cardiac output and blood volume due to the pathology of the pulmonary vasculature. Both pregnancy and delivery of a child exposes patients with PAH to the risk of right heart failure with the highest incidence of maternal mortality occurring during the first days after delivery (7). In addition to these pathophysiological threats, some of the commonly used PAH drugs are teratogenic. Thus, a secure contraception should be discussed, prescribed and repeatedly assessed in patients with advanced PAH. If unforeseen pregnancy occurs, early termination should be considered, which however does not go without risk either (6). Despite the enormous risks which pregnancy implicates in PAH for both, the patient and the fetus, some patients with mild symptoms and little impairment due to PAH in everyday life decide to become pregnant or to continue an incidental pregnancy. However, literature on the management and course of pregnancies in PAH is rare (1, 9, 10). The aim of the present retrospective study was to analyze outcomes of all pregnancies in women with PAH treated in Switzerland's largest expert center for pulmonary hypertension. More data on pregnancies in a diverse spectrum of patients with PAH are needed as a basis for better counseling women with PAH in childbearing age.

## Methods

This is a retrospective analysis of data from women diagnosed with PAH who conceived a pregnancy between the years 2004 and 2020 and were followed in the center for pulmonary hypertension at the University Hospital Zurich. Only patients diagnosed with PAH by RHC before pregnancy were eligible. All patients provided general consent for retrospective data analysis and the local ethics committee declared that no additional authorization is needed. The course during pregnancy in one patient (ID 4) was already published in a case report in 2009 (8).

Baseline data including demographics such as age, parity, classification of PAH, medication, co-morbidities, hemodynamics by RHC, echocardiographic values, World Health Organization functional class (WHO-FC) and 6-min walking distance (6MWD) were collected. The course of pregnancy was closely followed and hemodynamics by echocardiography including right ventricular function (fractional area change, tricuspid annular plane systolic excursion (TAPSE) and tricuspid regurgitation pressure gradient), blood N-terminal pro brain natriuretic peptide (NT-pro-BNP) and the WHO-FC were documented during the 1st, 2nd, and 3rd trimester as well as at follow-up's 3–5 months and 1–2 years after delivery as well as at the most recent follow-up. If the 6MWD was not available at follow-up, the peak work rate and oxygen uptake from ergospirometry were retrieved. Complications during pregnancy such as eclampsia, preeclampsia, thrombosis and worsening PAH related symptoms were noted. The method of delivery, anesthesia and perinatal complications as well as the condition of the neonates were documented, including neonatal death, small for gestational age (under the 10th customized centile) (11), preterm deliveries (prior to 37 weeks of gestation) (12), low birth weight ( ≤ 2,500 g) (13) and need for admission of the newborn to the neonatal intensive care unit. Well-controlled PAH was defined as patients in low risk profile under PAH targeted therapy (10).

### Data Presentation and Statistics

The different variables prior to, during and following pregnancy are descriptively presented as median (quartiles) or numbers (%). Statistic comparisons between different time points were performed by the Wilcoxon test. The calculations were made with SPSS 25 (SPSS, Chicago, IL, USA). A *p*-value <0.05 was considered significant.

## Results

### Patients and Baseline Characteristics

During the observational period, seven pregnancies in five women diagnosed with PAH occurred. In three women pregnancy was planned [ID 1 (2×), 2 and 3 (2×)] whilst two women became pregnant unplanned (ID 4 and 5). All of these pregnancies resulted in successful childbirth. Women with abortion would have been included as well, but no abortion occurred. All of these patients were regularly seen during pregnancy at the University Hospital Zurich, approximately once per month. Two patients gave birth twice and data from both pregnancies were analyzed (ID 1.1/1.2, and 3.1/3.2). One patient (ID 2) already had a child but her PAH diagnosis was made after her first pregnancy and herein only data from the second pregnancy was included. The other two women were nulliparous. Baseline characteristics of the last observation before pregnancy are shown in Table 1. The median maternal age at the beginning of the pregnancy was 29 (26; 36) years. Three patients were in WHO-FC I, three patients in WHO-FC II and one patient in WHO-FC III (ID 4). The latter was a women with PAH due to systemic lupus erythematosus with unplanned pregnancy and she was the only one with a PVR >4 WU before becoming pregnant. Three women were classified as idiopathic PAH whilst the last one had schistosomiasis associated PAH. The three women whose pregnancies were planned, were in the low-risk group according to risk stratification. We did not exclude patients with high risk PAH, but none of them became pregnant. This is likely because these women were firmly advised against pregnancy. The present collective of women with PAH who got pregnant was estimated to be <8% of women with PAH being in the childbearing age and followed at our center. Of interest, all three women revealed a >20% reduction of the PVR in vasoreactivity testing in the pre-pregnancy RHC and were prescribed CCB, albeit a formal >10 mmHg reduction of the mean PAP was not reached (but starting from a low mean PAP level). The most recent RHC was performed between 5 days to 20 months before pregnancy. For women with PAH who gave birth twice (ID 1 and 3), no RHC was performed between the two pregnancies. The median 6MWD was 630 (482; 750) m and the median measured NT-pro-BNP was 44 (41; 70) ng/l (norm <130 ng/l).

**Table 1:** Baseline characteristics before pregnancy onset.

Number of patients	5
Number of pregnancies	7
Age (years)	29 (27; 31)
Height (cm)	166 (165; 175)
Weight (kg)	55 (54; 66)
BMI (kg/m^2^)	20 (19; 21)
WHO-FC I/II/III	3 (43) /3 (43) / 1 (14)
1.1 Idiopathic PAH	3 (60)
1.4.1 PAH associated with connective tissue disease (systemic lupus erythematosus)	1 (20)
1.4.5 PAH associated with schistosomiasis	1 (20)
Heart rate at rest (bpm)	93 (75; 98)
SpO_2_ at rest (%)	97 (96; 100)
Blood pressure, systolic (mmHg)	114 (110; 130)
Blood pressure, diastolic (mmHg)	73 (72; 83)
6-minute walk distance (m)	630 (482; 750)
Heart rate after walk (bpm)	122 (108; 164)
SpO_2_ peak walk (%)	96 (95; 97)
NT-pro-BNP (ng/l)	44 (41; 70)
Right heart catheter before pregnancy onset (all patients on therapy)	
Mean pulmonary artery pressure (mmHg)	34 (28; 35)
Pulmonary artery wedge pressure (mmHg)	10 (10; 12)
Pulmonary vascular resistance (WU)	3.4 (2.4; 3.5)
Cardiac index (l/min/m^2^)	3.7 (3.6; 4.4)
SaO_2_ (%)	96 (94; 96)
SmvO_2_ (%)	73 (73; 80)
PaO_2_ (kPa)	11.0 (10.1; 12.4)
PaCO_2_ (kPa)	4.8 (4.6; 4.9)

*Data given as median (IQR) or number (% pregnancies)*.

*BMI, body mass index; WHO-FC, World Health Organization functional class; PH, pulmonary hypertension; SpO_2_, oxygen saturation; NT-pro-BNP, N-terminal pro brain natriuretic peptide; SaO_2_, arterial oxygen saturation; SmvO_2_, mixed venous oxygen saturation; PaO_2_, partial pressure of oxygen; PaCO_2_, partial pressure of carbon dioxide*.

### Course of the Pregnancy

**Figure 1:**
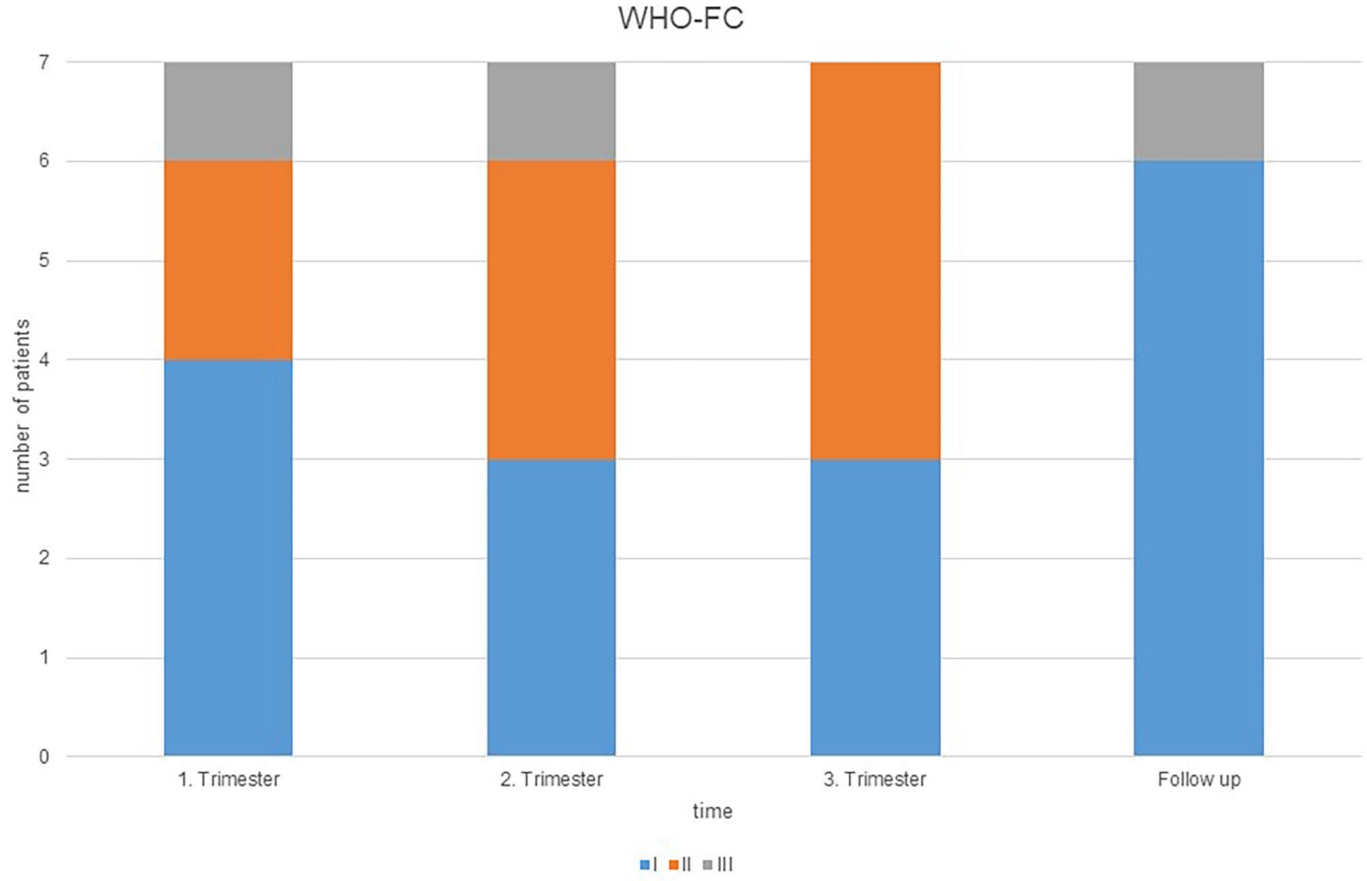
Course of the World Health Organization functional class (WHO-FC) during pregnancy and at the first follow-up 3–5 months after pregnancy.

The median pregnancy duration was 37 1/7 (37 0/7; 28 0/7) weeks. There were no clinical signs of worsening PAH during pregnancy. The course of echocardiographic data, NT-pro-BNP and WHO-FC before, during and after pregnancy are shown in Table 2. The changes of the WHO-FC are illustrated in Figure 1. There were no statistically significant changes in NT-pro-BNP that tended to increase in two and to decrease in five patients.

**Table 2:** Echocardiographic, laboratory and clinical data during and after pregnancy.

**Case**		**WHO-FC**	**TRPG (mmHg)**	**FAC(%)**	**TAPSE (mm)**	**NT-pro-BNP(ng/l)**	**REVEAL 2.0**	**ESC/ERS Score**
1.1	Before pregnancy	2	29	44	23	43	2	1
	1. Trimester	2	28	38	25	59		
	2. Trimester	2	25	36	26	65		
	3. Trimester	2	26	35	23	42		
	FU (3–5 months)	1	22	28	18	123		
	FU (1–2 years)	1	-	-	-	-		
1.2	Before pregnancy	1	22	28	18	123	2	1
	1. Trimester	1	30	40	20	41		
	2. Trimester	1	31	35	25	104		
	3. Trimester	2	39	37	28	96		
	FU (3–5 months)	1	40	41	25	136		
	FU (1–2 years)	1	37	36	20	77		
	Actual (3 years after)	1	35	28	26	48		
2	Before pregnancy	2	-	-	-	137	2	1
	1. Trimester	2	-	37	21	116		
	2. Trimester	2	41	44	28	78		
	3. Trimester	1	-	28	18	114		
	FU (3–5 months)	1	-	32	25	95		
	FU (1–2 years)	1	40	31	27	132		
	Actual (3 years after)	2	34	36	24	75		
3.1	Before pregnancy	2	41	26	17	44	3	1
	1. Trimester	1	21	41	22	93		
	2. Trimester	2	-	41	20	59		
	3. Trimester	2	41	36	21	34		
	FU (3–5 months)	1	41	32	20	67		
	FU (1–2 years)	1	-	37	18	30		
3.2	Before pregnancy	1	32	35	20	34	3	1
	1. Trimester	1	-	-	17	82		
	2. Trimester	1	28	33	18	-		
	3. Trimester	1	34	34	20	70		
	FU (3–5 months)	1	-	33	17	66		
	FU (1–2 years)	-	-	-	-	-		
	Actual (3 months after)	1	9	33	17	66		
4	Before pregnancy	3	49	18	17	70	4	1.1
	1. Trimester	3	-	55	-	80		
	2. Trimester	3	-	33	22	55		
	3. Trimester	2	40	31	15	35		
	FU (3–5 months)	3	57	45	23	242		
	FU (1–2 years)	3	55	51	18	98		
	Actual (15 years after)	3	45	35	11	99		
5	Before pregnancy	1	29	36	21	50	2	1.1
	1. Trimester	1	28	61	33	39		
	2. Trimester	1	-	41	26	61		
	3. Trimester	1	39	41	31	59		
	FU (3–5 months)	-	-	-	-	-		
	FU (1–2 years)	-	-	-	-	-		
	Actual (3 months after)	1	-	36	24	78		
Median	Before pregnancy	2 (1; 2)	30.5 (27.3; 43)	31.5 (24; 38)	19 (17; 21.5)	44 (41; 70)	2	1
(quartile)	1. Trimester	1 (1; 2)	28 (22.8; 29.5)	40 (37; 46)	21.5 (19.3; 27)	82 (59; 104)		
	2. Trimester	2 (1; 2)	28 (25; 28)	36 (33;41)	25 (20; 26)	63 (58; 82.5)		
	3. Trimester	2 (1; 2)	39 (26; 39)	34.5 (30.3; 36.3)	20.5 (17.3; 24.3)	50.5 (34.8; 81)		
	FU (3–5 months)	1 (1; 1)	40.5 (26.5; 53)	35 (27; 40)	21.5 (17.8; 25)	109 (66.8; 162.5)		
	FU (1–2 years)	1 (1; 2)	40 (37; 40)	36.5 (32.3; 47.5)	19 (18; 25.3)	77 (35.5; 115)		
	Actual (mean 4Y 3M)	2 (1; 3)	34 (9; 35)	35 (31; 36)	24 (14; 25)	75 (57; 89)		

*FU, follow up; TRPG, tricuspid regurgitation pressure gradient; FAC, fractional area change; TAPSE, tricuspid annular plane systolic excursion; WHO-FC, World Health Organization functional class; NT-pro-BNP, N-terminal pro brain natriuretic peptide. REVEAL 2.0 risk score (14); ESC/ERJ risk score (risk class of each parameter divided by available parameters) (15, 16)*.

*No significance before and after pregnancy was detected by Wilcoxon test for any value*.

Changes in drug therapy and detailed description of the medications of each woman before and during pregnancy as well as in childbed are shown in Table 3. In one patient (ID 2), the phosphodiesterase type 5 inhibitor was stopped during pregnancy because of headache and this patient additionally received diuretics due to leg edema. In another patient (ID 4), a CCB was given instead of a phosphodiesterase type 5 inhibitor as she reported, that she previously responded well to CCB, albeit formal vasoreactivity criteria were not fulfilled in the preceding RHC (3). In the two patients who were taking an endothelin receptor antagonist (ERA) when unintended pregnancy was discovered (ID 4 and 5), this medication was immediately stopped because of potential teratogenic effects (17, 18). The patient with ID 4 was switched to inhaled iloprost as she was in WHO-FC 3 before the discovery of the pregnancy, she had to stop the ERA and we wanted to keep her on dual combination therapy. The patients with schistosomiasis-associated PAH (ID 5) was switched from ERA to off-label oral selexipag after interdisciplinary discussion with the hospital pharmacist as this patient had an unfavorable hemodynamic profile at diagnosis, which responded very well under vasodilator combination therapy (reduction of the PVR from initially 9.2 to 2.5 WU before pregnancy onset) and we thus wanted to keep her on combination therapy In childbed, or in some cases already in the third pregnancy trimester, patients received prophylactic anticoagulation in form of subcutaneous low molecular weight heparin. Patient ID 4 was on vitamin K antagonist therapy at the time of discovery of pregnancy because of lupus-antigens, which was immediately stopped due to the known teratogenic effect and replaced by low molecular weight heparin (19).

**Table 3:** PAH targeted medication around pregnancy.

**Case**		**CCB**	**PDE-5**	**ERA**	**Anticoagulation**	**Diuretics**
1.1	Before	X	X			
(27 y/o)	During	X	X		X	
	Childbed	X	X		X	
1.2	Before		X			
(30 y/o)	During		X			
	Childbed		X		X	
2	Before	X	X			
(36 y/o)	During	X				X
	Childbed	X			X	
3.1	Before	X	X			
(26 y/o)	During	X	X			
	Childbed	X	X		X	
3.2	Before	X	X			
(31 y/o)	During	X	X			
	Childbed	X	X		X	
4	Before		X	X	X	X
(28 y/o)	During		X	^*^	X	X
	Childbed	X		^*^	X	
5	Before		X	X		X
(29 y/o)	During		X	^**^		X
	Childbed		X	^**^	X	

* *replacement of ERA with inhaled iloprost*.

** *replacement of ERA with selexipag*.

*y/o, years old; CCB, calcium canal blocker; PDE-5-inhibitor, phosphodiesterase type 5 inhibitor; s.c., subcutaneous; ERA, endothelin receptor antagonist; AC, anticoagulation*.

None of the women had severe pregnancy associated complications such as preeclampsia, eclampsia or thrombosis. In one patient (ID 2) an early pregnancy bleeding was present at 8 2/7 weeks of gestation. Therefore, progesterone was applied. In another pregnancy (ID 3.2), there was a rhesus constellation between mother and fetus. This was treated with maternal injection of an anti-D-prophylaxis.

### Perinatal Period and Delivery

In all cases, a cesarean section under regional anesthesia was recommended by our multidisciplinary team and successfully conducted in spinal anesthesia. Extended hemodynamic monitoring, including invasively measured blood pressure, central venous pressure and arterial and mixed-venous oxygen saturation was performed during delivery. All mothers gave birth to healthy babies. None of the neonates was small for gestation age or had low birth weight. After delivery, all mothers were observed in the intensive care unit for 1–2 days for safety reasons. Six sections were planned before term deliveries, with the latest at 38 0/7 gestational weeks. In one woman (ID 1.1) it was decided to terminate the gestation for safety reasons at week 33 5/7, whilst an extremely hot summer with temperatures over 35°C when the patient suffered from leg edemas and increased dyspnea. This patient had postpartal uterine bleeding under therapeutic anticoagulation on day two after delivery. That was treated only with fluid management and iron supplementation. Whilst this bleeding occurred, the patients was on the normal ward and was readmissioned to the intensive care unit for 1 day for safety observation without need for additional therapies.

### Follow-Up and Safety

Patients were regularly followed after pregnancy in the pulmonary hypertension center. Follow-up data of WHO-FC, NT-pro-BNP and echocardiography are shown in Table 2 and of 6MWD or cardiopulmonary exercise testing in Table 4. Symptoms returned back to pre-pregnancy levels and only the patient with PAH associated with systemic lupus erythematosus had slightly worse hemodynamics at follow-up. She had difficulties to perform exercise tests due to chronic knee pain. In all women, 6MWD 3–5 month after delivery was shorter compared to pre-pregnancy values, but in the majority, exercise capacity returned to pre-pregnancy values after 1–2 years.

**Table 4:** Six-min walking distance before and 3–6 month after pregnancy and at follow-up such as results of last cardiopulmonary exercise testing.

**Case**		**6-min walking distance (m)**	**Heart rate peak exercise (1/s)**	**Blood pressure end-exercise(mmHg)**	**SpO_**2**_ peak exercise (%)**	**Max. cycling, (Watt)**	**Peak VO_**2**_ (ml/kg/min)**
1.1	Before	630	108	123/76	95		
	After	542	93	97/72	94		
1.2	Before	630	104	114/73	97		
	After	586	73	112/66	81		
	16 months after	600	64	108/79	98		
	34 months after		175	181/79	91	215	29.1
2	Before	718	156	113/80	96		
	After	658	122	114/75	93		
	40 months after	708	146	125/74	96		
	43 months after		166	128	96	134	24.2
3.1	Before	784	171	154/78	96		
	After	780	175	-	96		
3.2	Before	750	164	124/81	95		
	After	-	-	-	-		
	4 months after		179	182/84	98	154	24.4
4	Before	454	108	120/60	95		
	After	414	114	110/60	99		
	177 months after	381	110	126/89	100		
5	Before	482	122	152/88	99		
	After	470	130	163/90	96		
	3 months after	470	130	163/90	96		
Median (quartile)	Before	^*^630 (482; 750)	114 (83; 148.5)	123/78(114/73; 152/81)	96 (95; 97)		
	After	^*^564 (456; 689)	118 (188; 141)	112/72(104/63; 139/83)	94 (88; 97)		

*FU, follow up; HR, heart rate; BP, blood pressure; SpO_2_, oxygen saturation*.

* *statistically significant, p<0.05 (Wilcoxon)*.

Table 5 summarizes obstetric characteristics, management, maternal and neonatal outcomes of all pregnancies.

**Table 5:** Characteristics and outcomes of the pregnancies.

**ID**	**WHO-FC**	**Parity**	**Etiology**	**PH severity**	**Delivery time (weeks)**	**Mode of delivery**	**Anesthesia**	**Weight newborn(g)**	**Maternal death**	**Neonatal death**
1.1	2	NP	Idiopathic	Mild	33 5/7	cs	Spinal	2,560	0	0
1.2	1	P	Idiopathic	Mild	37 0/7	cs	Spinal	3,510	0	0
2	2	P	Idiopathic	Mild	38 0/7	cs	Spinal	2,970	0	0
3.1	2	NP	Idiopathic	Mild	37 1/7	cs	Spinal	3,220	0	0
3.2	3	P	Idiopathic	Mild	37 1/7	cs	Spinal	2,880	0	0
4	1	NP	SLE	Moderate	37 1/7	cs	Spinal	2,760	0	0
5	1	NP	Schistosomiasis	Mild	38 0/7	cs	Spinal	3,500	0	0

*ID, identity; WHO-FC, World Health Organization functional class; PH, pulmonary hypertension; NP, nulliparous; P, parous; SLE, systemic lupus erythematosus; cs, cesarean section. PH severity defined as: mild, PVR < 4WU; moderate, PVR 4-6 WU*.

## Discussion

This case series summarizes seven successful pregnancies of women with well-controlled PAH who were stratified to be in the prognostic low-risk group. With one exception, patients were in WHO-FC I-II and had low NT-pro-BNP values. Hemodynamics were not severely impaired and PVR was <4 WU before pregnancy. All women were closely followed in our center, gave birth to healthy children and no serious adverse events or relevant worsening of PAH occurred (mean follow-up 4 years and 2 months). Compared to other series (9, 10, 20), all pregnant PAH-patients in in the present report were in a low risk group before pregnancy onset according to current guidelines or REVEAL scores (1).

During pregnancy, the body has to adapt to physiological changes affecting the cardiovascular system and several other organs. In PAH-patients, the pulmonary vascular disease impairs a physiologic vasodilator response. This may lead to increased pulmonary vascular resistance and the imminent risk of right heart failure (10, 21). Pregnancy may also uncover a previously undiagnosed PAH, as shown in a series from Sheffield, where 4/9 pregnant woman had a *de-novo* diagnosis of PAH discovered due to excessive dyspnea during pregnancy (9, 22). The management of pregnancies in PAH patients' needs a multidisciplinary team in a specialist center with experience in managing PAH as well as in complicated pregnancies and deliveries (9, 10, 20, 23). These women should be closely seen by experts and monitored for their disease as well as for fetus growth retardation (6, 24). The best follow-up strategy for pregnant patients with PAH is not known. Common risk assessments, such as the WHO-FC may be biased by increased dyspnea sensation which is caused by increased neural respiratory drive also seen in pregnancies in healthy women (25). Exercise tests by the 6MWD or ergospirometry are subject to the same bias. Due to the increased cardiac output in pregnancy, an increase in the tricuspid regurgitation pressure gradient is a physiological consequence and the same is true for right ventricular volumes, which has to be considered during echocardiographic follow-up. Common symptoms and signs of PAH, such as dyspnea, leg edema and fatigue, are also frequently found during pregnancy and distinction thus needs expertise (6, 9, 10, 26). The physiological adaptation to pregnancy may only be slightly impaired in patients with well-controlled PAH being in a good functional class and having only minor restrictions in physical activity. Good exercise tolerability before pregnancy onset may be a sign of preserved right ventricular contractile reserve and thus a beneficial indicator for favorable pregnancy outcomes. The paradigm of avoiding pregnancy for active women with well-controlled PAH may be associated with a very high psychosocial burden, lack of acceptance for these recommendations. Pulmonary hypertension centers face the dilemma of adherence to guidelines versus individualized patient counseling and provision of best follow-up care in difficult circumstances. In our collective, all patients wished to become mothers. In three patients, counseling was provided before contraception was stopped, in two of which, ERA was stopped and RHC was performed 3 months after stop in order to verify the absence of hemodynamic worsening. Two unplanned pregnancies occurred under potentially teratogenic ERA despite repeated counseling and prescription of contraceptive drugs.

In the current case series, all five women were on optimized PAH targeted medical treatment with 3/5 revealing borderline vasoreactivity in the most recent RHC before pregnancy. During pregnancy, these women did not reveal signs of PAH-worsening as assessed by WHO-FC, NT-pro-BNP or hemodynamics by echocardiography and thus, as in other series, RHC was not repeated (9). At echocardiography, right heart function was normal in all woman and only the woman with lupus-associated PAH with unplanned pregnancy was in a higher WHO-FC III, which even improved to II the third trimester, possibly related to a favorable immunomodulatory effect is systemic lupus erythematosus during pregnancy (27). However, her lupus worsened 11 weeks after delivery with affection of the hair, skin, joints and serosa resulting in a need to intensify immunosuppressive therapy.

In patients with PAH being in the intermediate to high risk group, labor, delivery and the post-partum period are associated with a high risk of mortality described up to 36% but with better outcomes in multidisciplinary PH-centers in recent years (1, 9, 10, 28, 29). As others, we recommended delivery by cesarian section in spinal anesthesia, as an expert team can be present, unforeseen hemodynamic threats during labor can be avoided (9, 10, 20, 30).

The first week of the post-partum period carries the greatest risk for maternal death (31). In a study published in 2014, 77 pregnancies were observed with an overall mortality of 16%, whereof 11% died in the post-partum period related to PAH, with the exception of one (22). An increase in cardiac output of up to 80% may be observed during this period due to auto-transfusion associated with uterine involution and the resorption of leg edema, which may lead to right ventricular failure and therefore, we observed PAH women for several hours at the ICU. Another risk in the post-partum period are thromboembolic events and therefore, prophylactic anticoagulation was started right after cesarean section in our patient collective (6). In all cases, a close monitoring is required during delivery and in the post-partum period (23).

The major limitation of this study is the small number of patients which got pregnant during the observational period. However, PAH is a rare disease and affected woman are generally counseled against pregnancy.

All pregnant women seen at our center between 2004 and 2021 had a favorable outcome, most probably due to their well-controlled PAH with low risk profiles managed by a multidisciplinary team in a reference center for pulmonary hypertension. We hope that our series together with others help treating physicians to counsel women with well-controlled PAH in the childbearing phase that do not wish abortion. However it has to be clearly stated that pregnancy may pose PAH-woman with less favorable risk profiles to considerable risk of disease progression and should thus be avoided.

## Data Availability Statement

The original contributions presented in the study are included in the article/supplementary material, further inquiries can be directed to the corresponding author.

## Ethics Statement

Ethical review and approval was not required for the study on human participants in accordance with the local legislation and institutional requirements. The patients/participants provided their written informed consent to participate in this study.

## Author Contributions

SU is the guarantor and takes responsibility for the content of the manuscript, including the data and analysis. NC, SU, and SS contributed to acquiring, analyzing, and interpreting the data, writing and revising the article critically for important intellectual content and providing final approval of the version to be published. CB, ML, ES, FG, AG, RS, and FK contributed to data collection, analysis and revising the article critically for important intellectual content. All authors take responsibility for all aspects of the reliability and freedom from bias of the data presented and their discussed interpretation. All authors contributed to the article and approved the submitted version.

## Conflict of Interest

SU reports grants from Swiss National Science Foundation, grants from Zurich Lung, grants from Orpha Swiss and Actelion SA, personal fees from Actelion SA, MSD, and Orpha Swiss, outside the submitted work. The remaining authors declare that the research was conducted in the absence of any commercial or financial relationships that could be construed as a potential conflict of interest.

## Abbreviations

PAH: pulmonary arterial hypertension
RHC: right heart catheter
PAP: pulmonary artery pressure
PVR: pulmonary vascular resistance
CCB: calcium channel blockers
6MWD: 6-min walking distance
FAC: fractional area change
TAPSE: tricuspid annular plane systolic excursion
TRPG: tricuspid regurgitation pressure gradient
NT-pro-BNP: N-terminal pro brain natriuretic peptide
ERA: endothelin receptor antagonist
WHO-FC: World Health Organization functional class.
